# A comprehensive evaluation of advanced dose calculation algorithms for brain stereotactic radiosurgery

**DOI:** 10.1002/acm2.14169

**Published:** 2023-09-29

**Authors:** Jihyung Yoon, Hyunuk Jung, Sean M. Tanny, Olga Maria Dona Lemus, Michael T. Milano, Sara J. Hardy, Kenneth Y. Usuki, Dandan Zheng

**Affiliations:** ^1^ Department of Radiation Oncology University of Rochester Medical Center Rochester New York USA

**Keywords:** acuros, AXB, eclipse, elements, Monte Carlo, SRS, VMC

## Abstract

**Purpose:**

Accurate dose calculation is important in both target and low dose normal tissue regions for brain stereotactic radiosurgery (SRS). In this study, we aim to evaluate the dosimetric accuracy of the two advanced dose calculation algorithms for brain SRS.

**Methods:**

Retrospective clinical case study and phantom study were performed. For the clinical study, 138 SRS patient plans (443 targets) were generated using BrainLab Elements Voxel Monte Carlo (VMC). To evaluate the dose calculation accuracy, the plans were exported into Eclipse and recalculated with Acuros XB (AXB) algorithm with identical beam parameters. The calculated dose at the target center (Dref), dose to 95% target volume (D95), and the average dose to target (Dmean) were compared. Also, the distance from the skull was analyzed. For the phantom study, a cylindrical phantom and a head phantom were used, and the delivered dose was measured by an ion chamber and EBT3 film, respectively, at various locations. The measurement was compared with the calculated doses from VMC and AXB.

**Results:**

In clinical cases, VMC dose calculations tended to be higher than AXB. It was found that the difference in Dref showed > 5% in some cases for smaller volumes < 0.3 cm^3^. Dmean and D95 differences were also higher for small targets. No obvious trend was found between the dose difference and the distance from the skull. In phantom studies, VMC dose was also higher than AXB for smaller targets, and VMC showed better agreement with the measurements than AXB for both point dose and high dose spread.

**Conclusion:**

The two advanced calculation algorithms were extensively compared. For brain SRS, AXB sometimes calculates a noticeable lower target dose for small targets than VMC, and VMC tends to have a slightly closer agreement with measurements than AXB.

## 1 INTRODUCTION

Stereotactic radiosurgery (SRS), as a highly precise and focal radiation treatment, has been a widely adopted modality to treat benign and malignant intracranial diseases such as meningioma, arteriovenous malformation (AVM), acoustic neuroma, trigeminal neuralgia, and limited brain metastases.^1^ In recent years, there has been increased utilization of SRS to treat multiple brain metastases, owing to comparable tumor control and patient survival as well as better cognitive function sparing compared with the conventional approach of whole brain radiotherapy.^2^, ^3^, ^4^ This change in practice pattern is largely attributable to technological advances in recent decades that have facilitated SRS for single and multiple targets. Better image guidance (e.g., with planar x‐rays, cone‐beam CTs, and surface tracking systems) has allowed non‐invasive frameless SRS to replace invasive frame‐based SRS,^5^, ^6^ which in turn allows for multi‐fraction SRS delivery which may be preferred over single‐fraction SRS in an effort to reduce toxicity risks.^7^ These imaging systems, with the support of a 6 degree‐of‐freedom couch, have also facilitated the use of a single‐isocenter to treat multiple brain metastases which increases the efficiency and cost‐effectiveness of treatment planning and delivery. Using high‐resolution multi‐leaf collimators (MLCs) with robust mechanical precision and flattening‐filter‐free modes with very high dose rates,^8^, ^9^ linear accelerator (LINAC)‐based and MLC‐based SRS has been increasingly adopted, making SRS widely accessible to more patients.

Facilitating these changes, vendors have released automated treatment planning modules and more advanced dose calculation algorithms for SRS planning. For MLC‐based SRS treatment planning on regular LINACs, the most current commercial solutions include the dynamic conformal arc (DCA) technique (Elements, BrainLab, Munich, Germany) and the volumetric arc therapy (VMAT) technique optimized either manually using conventional optimizers or semi‐automatically by HyperArc (Eclipse, Varian Medical Systems, Palo Alto, CA).^10^ The treatment of multiple small brain metastases with MLC‐based SRS, as opposed to cone‐based SRS, presents higher demands and challenges for dose calculation algorithm accuracy. Current advanced commercial dose calculation algorithms include Voxel Monte Carlo (VMC) available in Elements treatment planning system (BrainLab, Munich, Germany) and Acuros External Beam (AXB) available in Eclipse treatment planning system (Varian Medical Systems, Palo Alto, CA).^11^, ^12^

Dose calculation algorithms in radiotherapy treatment planning systems have progressed from correction‐based methods to model‐based methods (convolution methods), and most recently to Monte Carlo‐type algorithms.^13^ Among these advancements, tissue and material heterogeneity is increasingly better addressed to improve dose calculation accuracy. Based on different levels of accuracy, modern dose algorithms are often categorized into Type‐A (the least accurate algorithms such as pencil beam), Type‐B (the middle‐tier, currently standard‐of‐care, algorithms such as convolution superposition algorithms and the analytical anisotropic algorithm or AAA in Varian Eclipse), and Type‐C (the most accurate commercial algorithms such as VMC and AXB). Numerous studies have been published on implementing newer algorithms or on comparing the dosimetric accuracy of different types of dose algorithms.^14^, ^15^, ^16^, ^17^, ^18^, ^19^, ^20^ Comparisons have been reported both relative to each other and/or against measured dose results.^15^, ^16^, ^17^, ^18^, ^21^ As expected, the more advanced dose algorithms were found to yield more accurate dose calculations, especially in small‐field lung cases where tissue heterogeneity can be most influential. In these cases, target dose errors of up to 30% of the prescription have been reported for Type‐A algorithms, and those up to 15% have been reported for current standard‐of‐care Type‐B algorithms.^22^, ^23^, ^24^ In light of these results, lung SBRT protocols have required at least Type‐B algorithms in dose calculation and more advanced Type‐C dose algorithms have been introduced by commercial planning systems into clinical treatment planning.^25^ Currently, Type‐C dose algorithms in commercial treatment planning systems include the fast Monte Carlo algorithms such as BrainLab VMC and Elekta Monaco (Elekta AB, Stockholm, Sweden), and linear Boltzmann transport equation solver‐based algorithms Varian AXB. These algorithms are regarded as the most accurate clinical dose algorithms with target dose accuracy within 5% (or even 3%) with each other and with the gold‐standard full‐fledged Monte Carlo simulation.^26^, ^27^, ^28^, ^29^ For brain treatments, different dose algorithms have generally not been found to yield substantially different dosimetry. Nevertheless, brain SRS planning has also been upgraded to using the advanced Type‐C algorithms such as VMC and AXB in clinical practice.

In this article, we compare the dosimetry from the VMC and AXB dose calculation algorithms in the treatment of single or multiple targets, planned with a single isocenter. This novel and comprehensive evaluation includes a large number of patient cases and phantom comparisons with both ion chamber (IC) and film measurement results. Contrary to the commonly accepted beliefs that brain SRS is not sensitive to dose algorithms like thoracic SBRT, we show that there could be quantifiable differences between even two advanced dose algorithms in different treatment planning systems.

## 2 MATERIALS AND METHODS

### 2.1 Overall study design, simulation, and dose calculation

**FIGURE 1:**
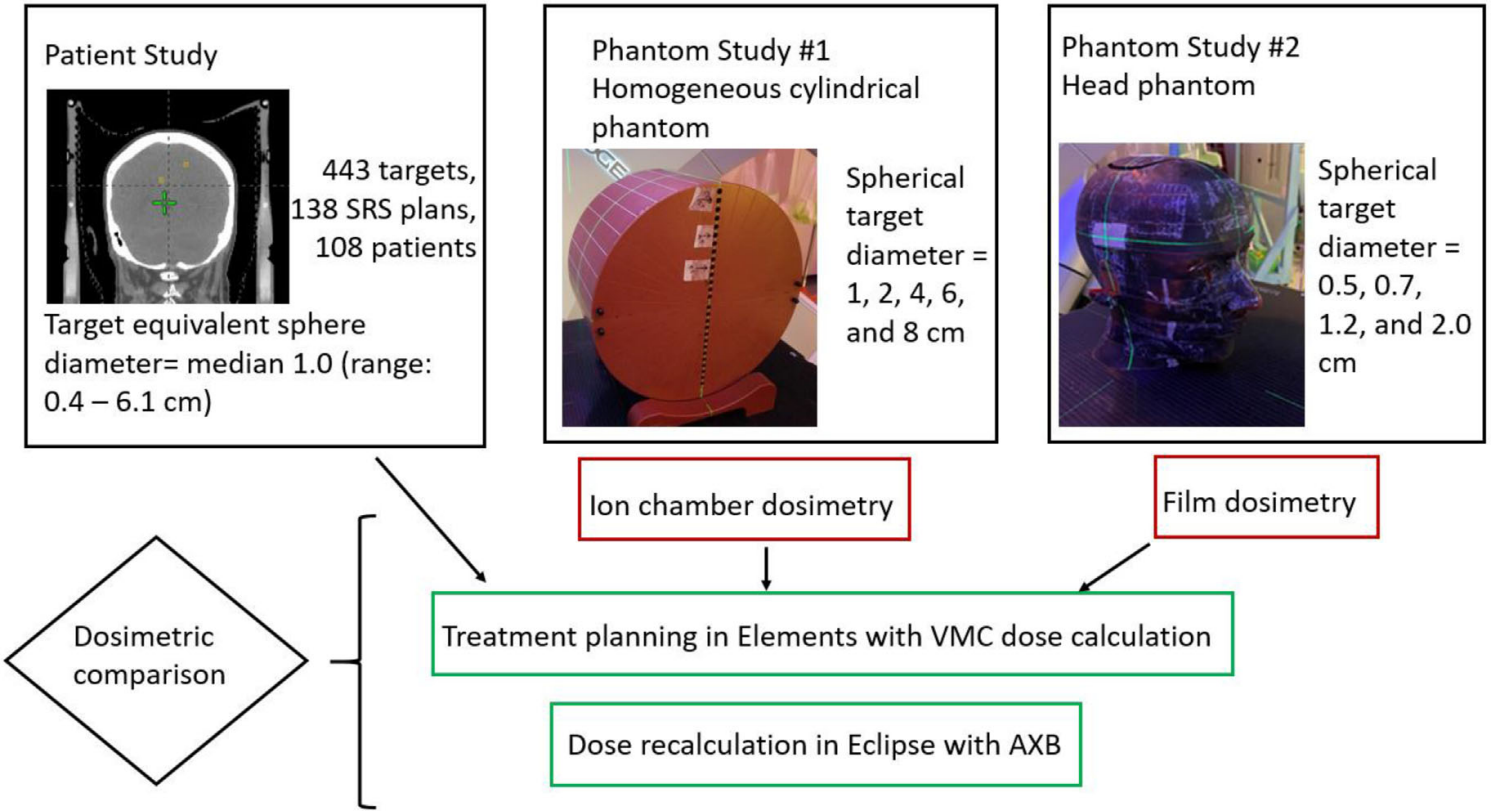
A schematic of the study design. The study compares VMC versus AXB dose for brain SRS in three investigations including a large‐cohort patient evaluation and two phantom evaluations. In the cylindrical phantom study with various target sizes, the two calculated doses were benchmarked against ion chamber measured dose at isocenter and off‐axis. In the head phantom study with various target sizes and target locations, the two calculated doses were benchmarked against film dosimetry.

Two advanced algorithms, the Voxel Monte Carlo (VMC) available in Elements treatment planning system (BrainLab, Munich, Germany), and the Acuros External Beam (AXB) available in Eclipse treatment planning system (Varian Medical Systems, Palo Alto, CA), were compared and benchmarked with measured doses in various conditions in this study. As illustrated in Figure 1, this study included three investigations. In the patient study, the calculated doses were retrospectively compared with the two different dose algorithms on a large cohort of patients treated with SRS. This study was approved by the institutional Human Subjects Review Board. In phantom study #1, using a homogeneous cylindrical phantom, the calculated dose was compared with ion chamber dosimetry measured at the central axis and off‐axis positions for a range of small‐to‐large target sizes. In phantom study #2, using a heterogeneous anthropomorphic phantom, the calculated dose was compared with film dosimetry measured with EBT3 film for a range of small‐to‐medium target sizes and different target locations (four target sizes and three different target locations).

The patient and phantoms were scanned with a GE LightSpeed RT 16 CT scanner (GE Healthcare, Little Chalfont, UK) with a slice thickness of 1.25 mm. The images were imported into BrainLab Elements for the plan generation with VMC calculation. To obtain AXB‐calculated doses, the generated plan in Elements was subsequently exported into Eclipse for AXB dose re‐calculation using identical beam parameters including MLC motions and monitor units. Therefore, the difference in plan optimization algorithms could be ignored in this study. The dose grid size was 1 mm in AXB recalculation, which is the finest resolution in Eclipse, where it was the same to CT image resolution (0.8 × 0.8 × 1.3 mm^3^ in this study) in Elements. In this way, the difference in dose calculation can be directly compared without accounting for the difference in optimization algorithms of different planning systems. All plans used a Varian Edge LINAC with HD‐MLCs (Varian Medical Systems, Palo Alto, CA), and our institutional clinical practice of prescription dose to at least 95% of planning target volume (PTV) was adopted (PTV D95≥ 100% of prescription dose).

### 2.2 Retrospective patient study

The treatment plans from 108 consecutive patients (138 SRS plans with a total of 443 targets) who received SRS at our clinic from August 2021 to February 2022 were evaluated. These cases included both primary and metastatic lesions and were planned in BrainLab Elements using volumetric modulated arc therapy (VMAT) for single‐target cases and dynamic conformal arc (DCA) for single‐isocenter‐multiple‐target cases, prescribed for 27 Gy/3fxs. The original VMC dose was imported into Eclipse and subsequently compared with the recalculated AXB dose, focused on the targets using the following dosimetric endpoints: the reference dose at the individual PTV center (Dref), dose received by 95% of the PTV volume (D95), and mean PTV dose (Dmean). Per our departmental clinical practice, SRS cases are prescribed such that PTV D95 ≥ 100% of the prescription dose. A Dref difference of 5% was used as the action threshold to assess the potential dependence of target dose difference on several targets and plan characteristics, based on the recommendation that the dose to be delivered to within 5% of the prescribed dose by the International Commission on Radiation Units and Measurements.^30^ The studied factors include target volume, planning technique (VMAT vs. DCA), target‐to‐isocenter distance, and target‐to‐skull distance. The distance of target‐to‐skull was manually measured in Eclipse by rotating CT images to find the closest skull point from the target center for 11 randomly chosen patients with 47 targets, and the collected data were analyzed to find any possible trends.

### 2.3 Phantom study #1 – homogeneous phantom

A homogeneous cylindrical phantom of virtual water density (the TomoTherapy “cheese” phantom) was used for virtual SRS planning with target sizes and ion chamber dose measurements. The phantom has a diameter of 30 cm and a length of 18 cm. A single virtual spherical PTV of six varying diameters between 0.6 and 8 cm was placed at the center of the phantom. A single‐target VMAT SRS treatment plan was developed using BrainLab Elements Cranial SRS v3.0 (BrainLab, Munich, Germany), calculated with its VMC dose algorithm and prescribing 6 Gy to the PTV. Three 160‐degree arcs with a 30‐degree collimator rotation at couch angles of 350, 0, and 15 degrees were used for all plan optimizations in BrainLab Elements. The optimized plans were exported into Eclipse v15.6 (Varian Medical Systems, Palo Alto, CA) and recalculated with its AXB dose algorithm.

**FIGURE 2:**
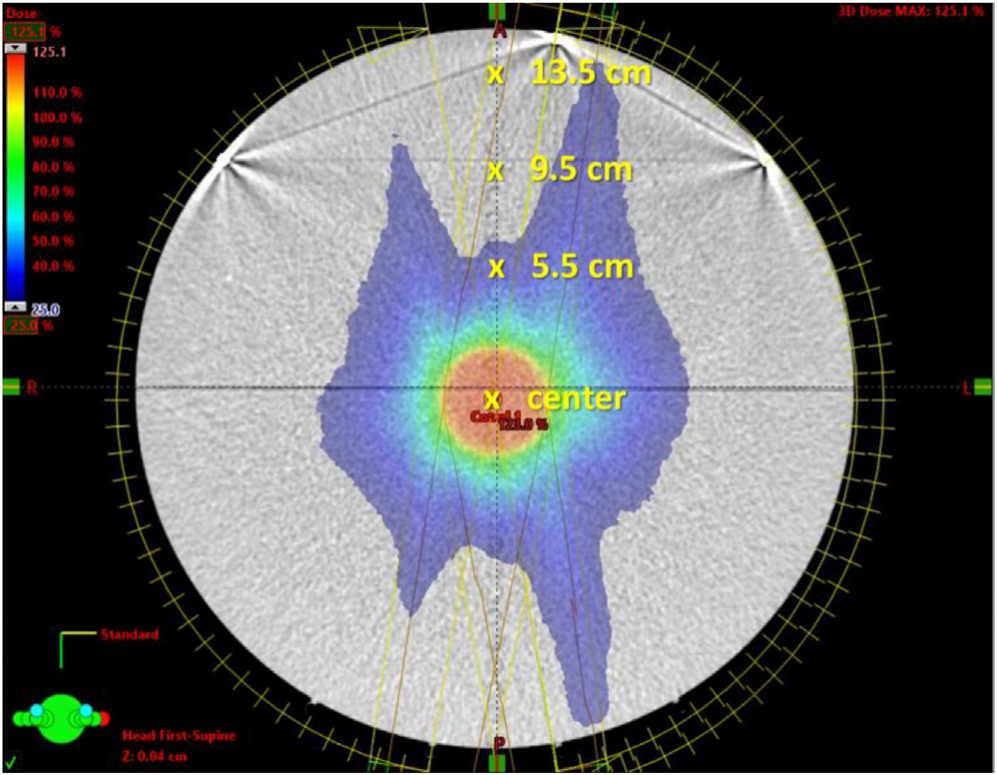
The cylindrical phantom and measurement points used in phantom study #1. The example plan shown was for a PTV of 4 cm diameter.

For each of the five plans, absolute dose measurements were performed using an Exradin A1SL ion chamber (active volume of 0.053 cm^3^) at the isocenter and off‐axis points at 5.5, 9.5, and 13.5 cm as shown in Figure 2. To minimize the impact from linac daily variation, the IC measurements were normalized to a solid‐water phantom at 1.5 cm depth, 10 × 10 cm^2^, with 100 monitor units (MU) giving 1 Gy. To calculate dose from VMC and AXB, the effective volume of the ion chamber was contoured at each measurement point in BrianLab Elements, and the mean dose for the volume was obtained from each treatment planning system.

### 2.4 Phantom study #2 – anthropomorphic phantom

**FIGURE 3:**
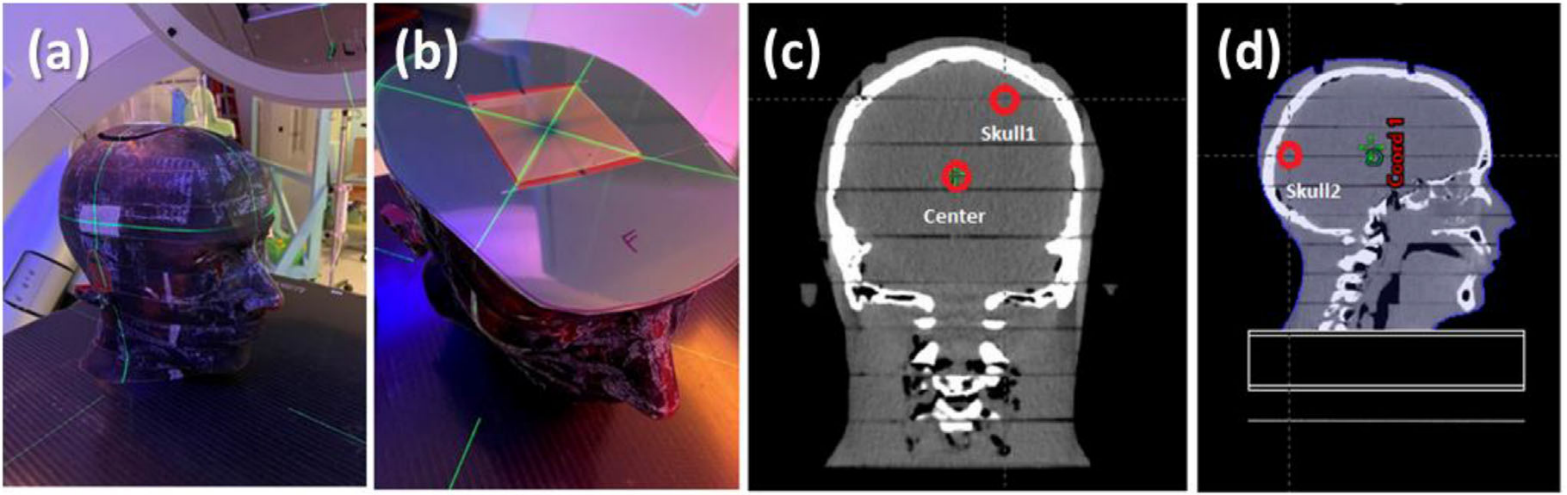
Experiment setup for phantom study #2: (a) the head phantom used in this study, (b) film placement for one centrally‐located PTV, (c) and (d) PTV locations in this experiment.

A head phantom was used in the second phantom investigation with film dosimetry. The phantom was placed in an upright position on the table (Figure 3a) to minimize the air gap between phantom slabs and the film and also to improve setup reproducibility. Also, a dummy film template was placed around the measurement film (Figure 3b) to fill the air gap between the slabs.

Similar to described in Section 2.3, a single‐target SRS plan was developed using BrainLab Elements on a virtual spherical PTV of four varying diameters between 0.5 and 2 cm. For each instance, the target location was varied at the center of the brain and two near‐skull locations to assess the effect of phantom heterogeneity (Figure 3c, d). Note that the figures were generated to show all target positions at once, but in each case, a single target was planned and measured. Dose recalculation was performed in Eclipse with AXB for comparison.

For each of the 12 plans (four target sizes and three target locations), film dosimetry was performed using Gafchromic EBT3 film (Ashland Advanced Materials, Bridgewater, NJ). For each plan, a single measurement was acquired while for the calibration curve, three repeat measurements were taken for each point. The irradiated films were scanned with Epson Expression 11000XL and analyzed with ImageJ (National Institutes of Health, Bethesda, MD) and an in‐house MATLAB (Math‐ Works Inc., Natick, MA) script. The film and calculation results were quantitatively compared by field width at the 100% and at 50% prescription levels as well as the slope (dose gradient) in 25%−100% prescription level (about 20%−80% of Dmax).

### 2.5 Additional dose calculation comparison for small fields

Open square field output doses were calculated to investigate the impact of MLC modeling in Eclipse and Elements. Square field sizes from 1 × 1 to 4 × 4 cm^2^ were tested with Jaws (MLCs fully retracted) or MLC (jaws were 2 mm retracted beyond MLC on each side). The 2 mm jaw offset is based on the Elements parameter in our clinic and is also the global setting provided by BrainLab.

Additionally, doses were calculated with very small MLC fields of 0.5 × 0.5 and 0.2 × 1.0 cm^2^. The corresponding jaw sizes were 1.0 × 1.0 and 1.0 × 1.4 cm^2^, respectively. These conditions were selected to simulate realistic scenarios for DCA and VMAT plans generated by Elements during clinical beam delivery.

All simulations were conducted at a source‐to‐surface distance (SSD) of 100 cm with 6 FFF beam and 300 MUs. Point doses were compared at depths of 1.5, 5, 10, and 15 cm between the two Treatment Planning Systems (TPS).

## 3 RESULTS

### 3.1 Retrospective patient study

**FIGURE 4:**
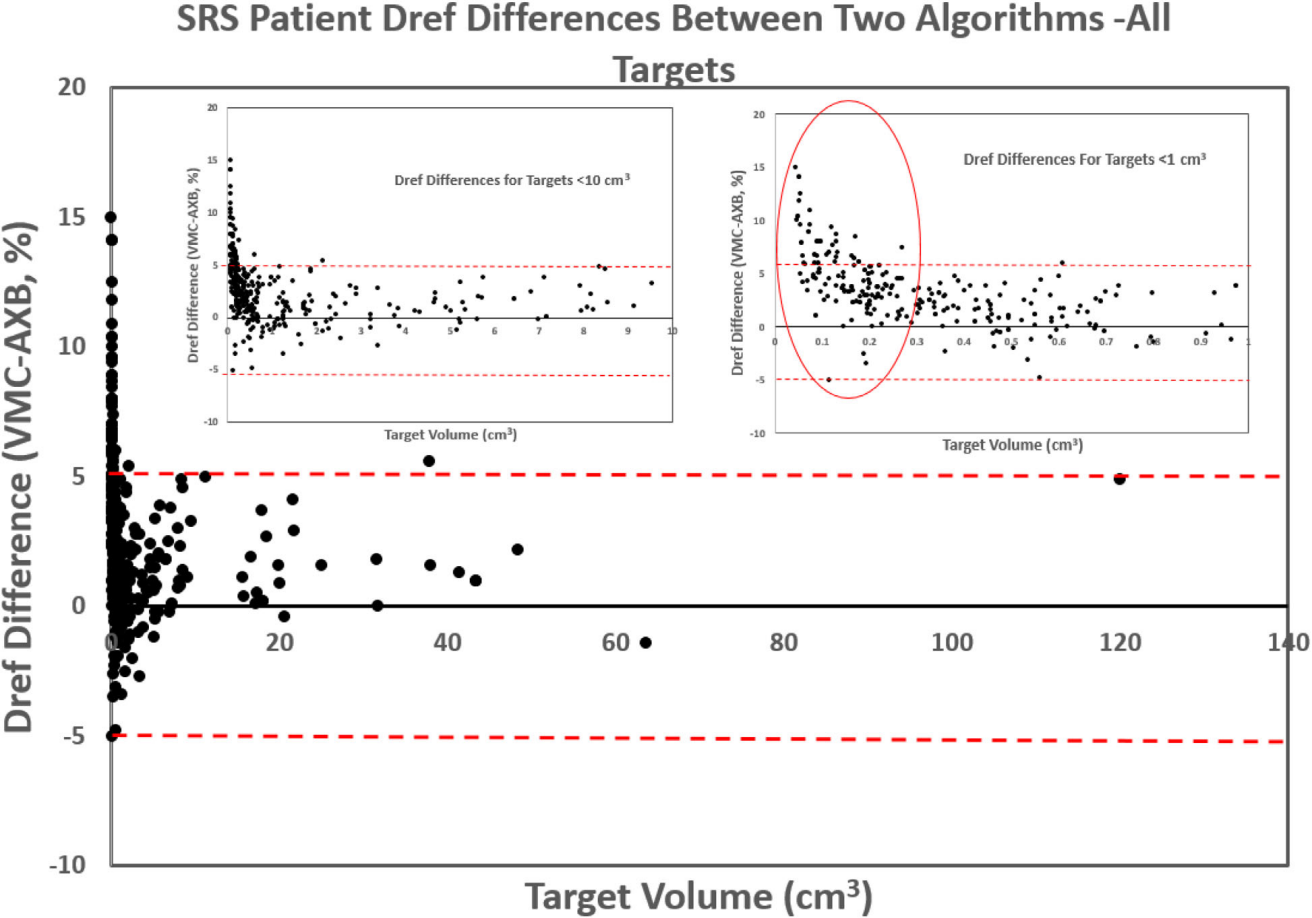
Dose differences at Dref between the two algorithms plotted for all 443 targets. When zoomed in (to <10 cm^3^ in the upper left insert and <1 cm^3^ in the upper right insert), a target size dependency can be observed where larger dose differences tend to associate with smaller target sizes.

The median volume of the 443 targets included in the patient study was 0.5 cm^3^ (range: 0.04–120 cm^3^). Comparing the two algorithms, VMC tends to calculate Dref higher than AXB, as shown in Figure 4 with most data points having positive values, with an average difference of 2.3%. However, some targets also calculated very large differences between the two algorithms, leading to a large standard deviation of 2.6% in the sample. Of the 443 targets, 53 targets (12%) showed ≥ 5% Dref differences between the algorithms (maximum = 15%). These targets came from cases including 10 single‐target VMAT plans and 15 single‐isocenter‐multiple‐target DCA plans. No difference was found between the two algorithms in terms of target dose discrepancies. Figure 4 plots the calculated Dref difference between the two algorithms (VMC‐AXB) versus target volume. It can be seen from the plots that as the target size gets smaller, the Dref difference has a wider spread with an increasing number of cases showing ≥5% Dref differences. It was found that differences in Dref ≥5% tend to occur with smaller (< 0.3cm^3^) targets.

**FIGURE 5:**
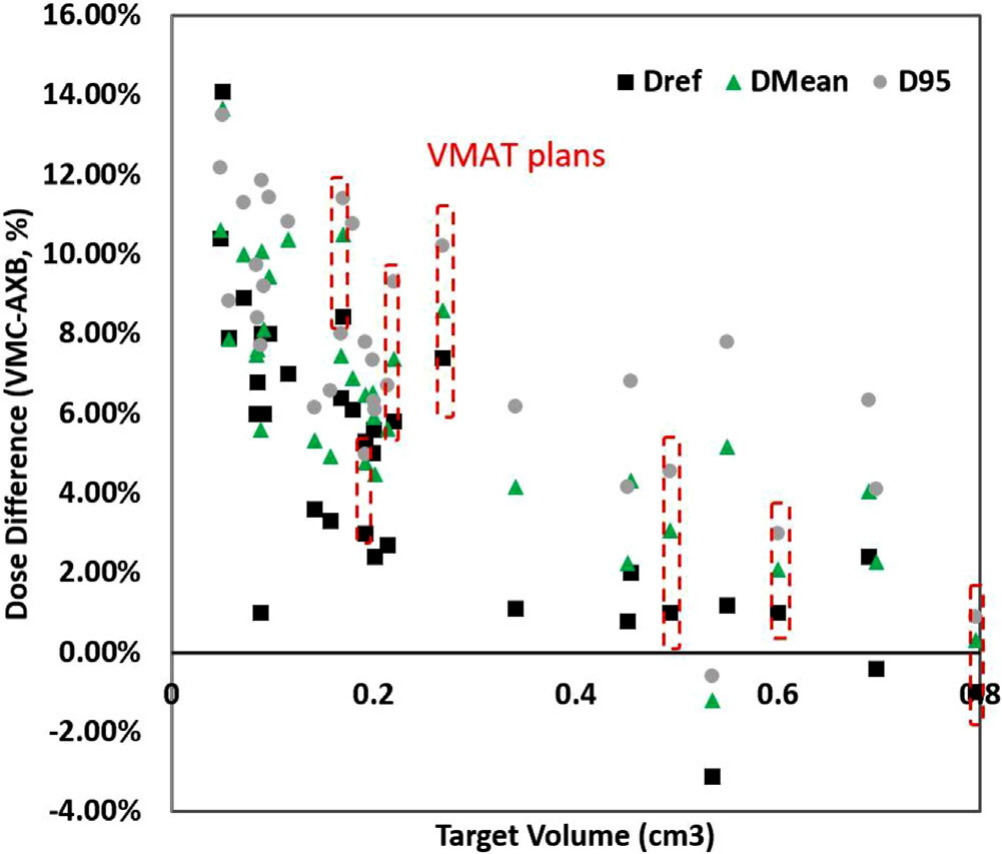
Differences of Dref, D95, and Dmean plotted against target volume, for example, cases to show a dependency with target size for all endpoints, independent of planning technique.

**FIGURE 6:**
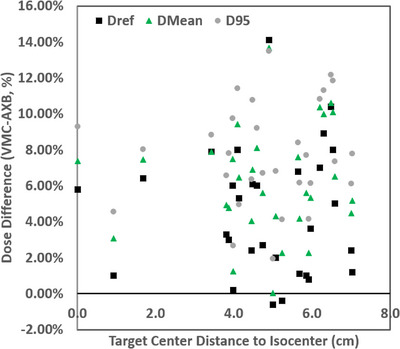
Differences of Dref, D95, and Dmean plotted against the distance between the isocenter and the target center, for example, cases to show a lack of dependency with the distance to the isocenter.

The discrepancies between the two planning algorithms in Dref, D95, and Dmean versus target volume for a subset of randomly selected 36 targets are shown in Figure 5. The magnitudes of D95 and Dmean discrepancies are often even higher than those of Dref. And similarly, D95 and Dmean differences also tend to be higher for smaller target sizes. The trend was observed for both single‐target VMAT cases and multi‐target DCA cases. The target dose differences were also plotted in Figure 6 against the distance between the isocenter and the target center. No observable trend was found.

**FIGURE 7:**
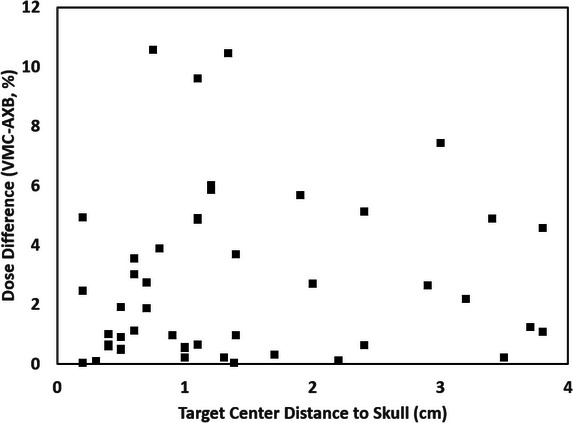
Differences of Dref are plotted against the distance between the target center and the closest skull surfaces, for example, cases to show a lack of dependency with the distance to the skull.

Dref differences for example targets were also plotted against the measured distance between the target center and the closest skull surface in Figure 7. No apparent trend was observed between target dose differences and the distance to the skull.

No correlation between target‐to‐isocenter distance, technique (VMAT vs. DCA), or heterogeneity (target‐to‐skull distance) with the observed dose differences between VMC and AXB. However, we observed a strong correlation for target size, where there is a higher chance of having a large target dose difference for smaller targets. A threshold of 0.3 cm^3^ was selected based on the data trend which identified an odds ratio of 41.1 (95%CI = 14.5–116.2, *p* < 10^−10^) on Dref > 5% for target volume < 0.3 cm^3^ versus ≥0.3 cm^3^ (corresponding to an equivalent sphere diameter of 8.3 mm).

### 3.2 Phantom study #1: Homogeneous phantom

Table 1 summarizes the homogeneous cylindrical phantom investigation. At the central axis (isocenter), for all but the smallest PTV with a 0.6 cm diameter, calculated doses from both dose algorithms were in close agreement with the actual delivered dose measured with the ion chamber with a maximum difference of 2.1%. VMC and AXB calculations were slightly lower than the measured dose by −0.7% and −1.6% on average, respectively. For the smallest PTV, a fairly large dose difference (−13.1%) was observed for AXB at the isocenter, while VMC agreed well with the IC measurement (1.9%).

**TABLE 1:** Dose difference in absolute and relative values for each algorithm in phantom study #1.

		Center			5.5 cm			9.5 cm			13.5 cm		
PTV diameter (cm)		Rx level (%)	Abs. (cGy)	Rel. (%)	Rx level (%)	Abs. (cGy)	Rel. (%)	Rx level (%)	Abs. (cGy)	Rel. (%)	Rx level (%)	Abs. (cGy)	Rel. (%)
0.6	VMC	117.5	65.5	1.9	0.7	−1.3	−6.4	0.2	0.7	10.0	0.1	−0.3	−9.8
	AXB		−460.7	−13.1		−0.3	−1.4		−0.5	−7.6		−0.5	−16.2
1	VMC	115.6	−72.3	−2.1	1.9	4.0	7.1	0.5	−0.6	−4.3	0.2	0.8	15.0
	AXB		−62.5	−1.8		−7.2	−12.9		−3.9	−27.8		−1.3	−26.7
2	VMC	109.3	−20.0	−0.6	9.8	21.4	7.3	1.2	0.8	2.3	0.4	0.0	0.3
	AXB		−56.4	−1.7		−8.3	−2.8		−6.9	−19.2		−4.0	−31.3
4	VMC	121.6	−26.2	−0.7	32.3	25.6	2.6	9.6	3.6	1.2	1.3	9.4	25.0
	AXB		−40.3	−1.1		−12.8	−1.3		−12.6	−4.4		0.7	1.9
6	VMC	121.8	−16.4	−0.4	54.2	29.1	1.8	25.9	9.8	1.3	9.5	8.2	2.9
	AXB		−65.3	−1.8		−12.7	−0.8		−10.8	−1.4		2.5	0.9
8	VMC	119.8	16.8	0.5	66.9	25.1	1.3	36.3	15.2	1.4	18.3	26.8	4.9
	AXB		−53.0	−1.5		−34.0	−1.7		−28.0	−2.6		4.7	0.9

**FIGURE 8:**
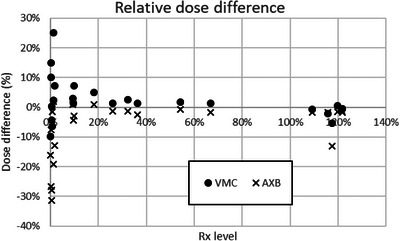
Dose difference from ion chamber measurements by prescription level in relative scale for phantom study #1.

At the off‐axis measurement points, both algorithms yielded dose differences greater than 5% compared with the measured dose, but only in the low dose region at below 20% of the prescription level (Figure 8 and Table 2), similar to what was previously reported for these commercial treatment planning algorithms.^31^, ^32^, ^33^ VMC calculations were higher than measurements in general, while AXB calculations were lower than measurements.

**TABLE 2:** Profile comparisons from each TPS calculation to the film measurement.

		PTV 5 mm			PTV 7 mm			PTV 12 mm			PTV 20 mm		
Location		AXB	VMC	Film	AXB	VMC	Film	AXB	VMC	Film	AXB	VMC	Film
Center	Rx width (diff)	0.313	0.401	0.487	0.462	0.592	0.631	1.110	1.184	1.242	1.943	1.973	1.954
		(−0.174)	(−0.086)		(−0.169)	(−0.039)		(−0.133)	(−0.059)		(−0.010)	(0.019)	
	FWHM (diff)	8.642	8.927	8.636	10.926	11.190	10.859	17.024	17.453	17.293	25.910	26.540	26.204
		(0.006)	(0.291)		(0.067)	(0.332)		(−0.269)	(0.160)		(−0.294)	(0.335)	
	Slope (diff)	17.358	19.422	24.606	15.703	18.007	20.721	15.519	15.909	18.032	12.810	12.327	12.955
		(−7.248)	(−5.184)		(−5.018)	(−2.714)		(−2.513)	(−2.123)		(−0.145)	(−0.628)	
Skull1	Rx width (diff)	0.317	0.401	0.491	0.510	0.630	0.582	1.104	1.232	1.215	1.921	2.004	2.036
		(−0.175)	(−0.090)		(−0.072)	(0.048)		(−0.111)	(0.017)		(−0.115)	(−0.032)	
	FWHM (diff)	9.186	8.927	9.144	11.320	11.603	11.176	17.369	17.831	17.695	25.659	26.450	26.501
		(0.042)	(−0.217)		(0.144)	(0.427)		(−0.326)	(0.136)		(−0.842)	(−0.051)	
	Slope (diff)	16.328	19.422	21.475	15.866	17.237	18.124	14.393	15.437	16.558	12.348	12.172	13.172
		(−5.146)	(−2.052)		(−2.259)	(−0.887)		(−2.164)	(−1.121)		(−0.824)	(−1.000)	
Skull2	Rx width (diff)	0.375	0.477	0.506	0.509	0.680	0.665	1.167	1.260	1.257	2.002	2.061	2.062
		(−0.131)	(−0.029)		(−0.155)	(0.015)		(−0.091)	(0.003)		(−0.060)	(−0.001)	
	FWHM (diff)	9.057	9.535	9.123	11.308	11.800	11.303	17.129	17.903	17.420	25.696	26.311	26.077
		(−0.066)	(0.412)		(0.005)	(0.497)		(−0.292)	(0.482)		(−0.382)	(0.234)	
	Slope (diff)	17.657	19.536	22.641	16.095	18.292	20.019	15.695	16.127	17.284	13.142	13.152	13.296
		(−4.984)	(−3.105)		(−3.924)	(−1.726)		(−1.590)	(−1.158)		(−0.153)	(−0.143)	

Unit = mm (Rx width and FWHM) and %/mm (slope).

### 3.3 Phantom study #2: Anthropomorphic phantom

**FIGURE 9:**
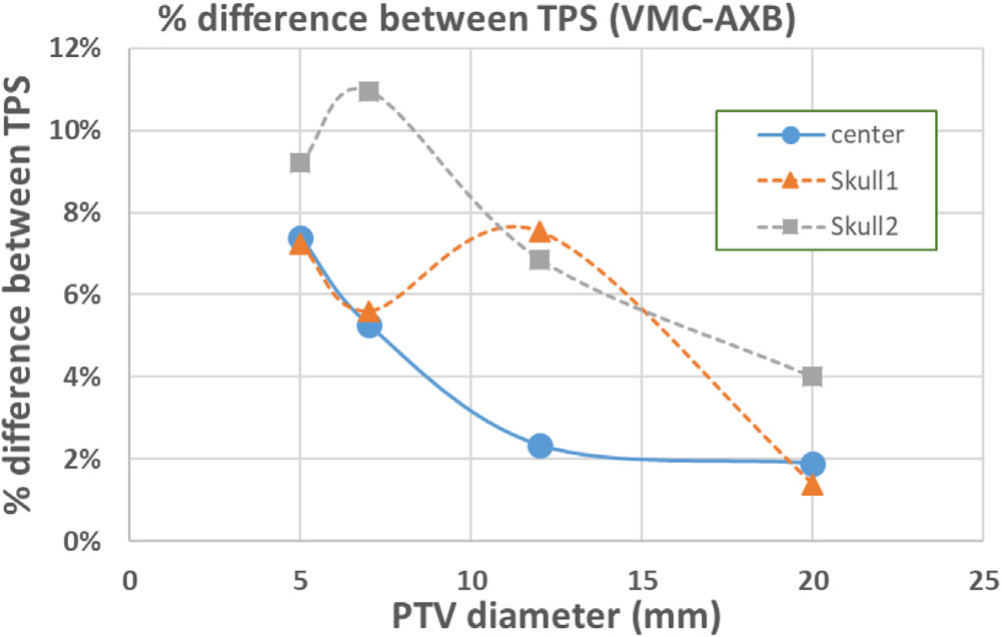
The dose difference at the center of PTVs between VMC and AXB in phantom study #2 (head phantom). The corresponding PTV volumes are 0.07, 0.18, 0.90, and 4.19 cm^3^, from left to right.

Phantom study #2 using the head phantom compared the calculated and measured target dose for small‐to‐medium sized targets. Figure 9 plots the calculated isocenter dose differences between the two dose algorithms for the 12 studied cases. The VMC dose tends to be higher than the AXB dose, and greater than 5% dose differences were found between the two dose algorithms for small PTVs. At the more central and away‐from‐skull location (denoted as “center”), >5% dose differences were observed for PTV diameters of 5 and 7 mm (corresponding to 0.07 and 0.18 cm^3^ in volumes, respectively). For the two near‐skull locations, in addition to PTV diameters of 5 and 7 mm, >5% dose differences were also observed for a PTV diameter of 12 mm (corresponding to 0.90 cm^3^ in volume). For the central location test cases, the difference between algorithms decreased as the PTV size increased. For near‐skull cases, there was no monotonic trend, but the two algorithms agreed within 5% for the largest PTV at a diameter of 20 mm (Figure 9).

**FIGURE 10:**
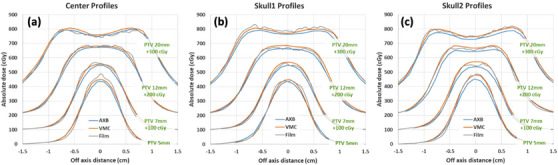
Measured versus calculated dose profiles at (a) the center, (b) and (c) near the skull for phantom study #2 (head phantom). Arbitrary numbers were added for renormalizing the dose results to distinguish profiles with different PTV diameters (green descriptions in the plots). While the measured profiles contain higher noise, overall it seems the film measurements showed somewhat closer agreement with VMC calculations.

The film‐measured dose profiles are plotted in Figure 10, reflecting the actual delivered dose, along with calculated dose profiles for comparison. The delivered dose measurement on film was noisy due to limitation of the film dosimetry, but a slightly closer agreement was observed between the measurement and VMC than AXB, especially for the high dose spread region (i.e., around the prescription level) and the dose gradient region. The discrepancy between the measured and calculated dose was also quantified as shown in Table 2. Field width at the 100% and at 50% prescription levels as well as the slope (dose gradient) in 25%−100% prescription level (about 20%−80% of Dmax) were listed. VMC was found to have smaller differences than AXB when compared to the film measurements, in the high dose region (field width at the 100% prescription level) and in dose gradient.

### 3.4 Additional dose calculation comparison for small fields

The square field comparison results are listed in Table 3. The results show that AXB and VMC exhibit distinct trends. For AXB, there was minimal difference between MLC‐defined fields and jaw‐defined fields for all field sizes. In contrast, in VMC, MLC‐defined fields generally result in higher doses, also as field size decreases, the difference between MLC‐defined fields and jaw‐defined fields becomes more pronounced. For a field size of 1 × 1 cm^2^, VMC calculations from MLC‐defined field are higher than jaw‐defined field by 6.6% on average.

**TABLE 3:** Dose calculations of open square fields by field sizes.

Field size	Depth (cm)	Jaw‐defined field (cGy)					MLC‐defined field (cGy)				
		MLC	Jaw	AXB	VMC	diff (%)	MLC	Jaw	AXB	VMC	diff (%)
1 × 1 cm^2^	1.5	Open	1 cm	252.9	246	2.8	1 cm	1.4 cm	251.9	261	−3.5
	5			195.7	191	2.5			194.9	203	−4.0
	10			135.5	130	4.2			134.9	140	−3.6
	15			95.1	93	2.3			94.6	99	−4.4
2 × 2 cm^2^	1.5		2 cm	275.2	281	−2.1	2 cm	2.4 cm	275.1	284	−3.1
	5			219.3	220	−0.3			219.1	222	−1.3
	10			153.4	157	−2.3			153.2	155	−1.2
	15			108.2	109	−0.7			108.1	108	0.1
3 × 3 cm^2^	1.5		3 cm	280.9	280	0.3	3 cm	3.4 cm	281.1	288	−2.4
	5			228.2	231	−1.2			228.4	230	−0.7
	10			161.1	159	1.3			161.2	163	−1.1
	15			114	111	2.7			114.1	114	0.1
4 × 4 cm^2^	1.5		4 cm	284.6	290	−1.9	4 cm	4.4 cm	285	292	−2.4
	5			234.2	236	−0.8			234.5	238	−1.5
	10			167.1	169	−1.1			167.3	171	−2.2
	15			119	120	−0.8			119.1	123	−3.2

Additionally, at a 1 × 1 cm^2^ field size, VMC calculations were lower than AXB in jaw‐defined field, whereas they were higher in MLC‐defined field. The difference between AXB and VMC in MLC‐defined field was approximately 4%.

In Table 4, the differences between the two algorithms are listed for two example very small MLC fields. For MLC‐defined field of 0.5 × 0.5 cm^2^, the difference was over 6%. For 0.2 × 1.0 cm^2^ MLC opening, the difference was significantly increased to approximately 15%.

**TABLE 4:** Dose calculation for very small MLC defined fields.

Jaw	MLC	depth (cm)	AXB (cGy)	VMC (cGy)	diff (%)
1.0 × 1.0	0.5 × 0.5	1.5	196.4	211	−6.9
		5	150.3	160	−6.1
		10	102.4	109	−6.1
		15	70.5	77	−8.4
1.0 × 1.4	0.2 × 1.0	1.5	137.8	163	−15.5
		5	105.6	123	−14.1
		10	72.2	84	−14.0
		15	49.9	59	−15.4

## 4 DISCUSSION

Advanced dose calculation algorithms were compared from two major treatment planning systems widely used for MLC‐based SRS on regular LINACs. For brain SRS, it is conventionally believed that the largely homogeneous brain tissue makes it not as sensitive to dose algorithms, and less advanced algorithms have for a long time been considered acceptable. Correction‐based methods such as the water‐based TMR classic algorithm (GammaPlan, Elekta AB, Stockholm, Sweden) still are used for SRS planning. On the other hand, all major treatment planning vendors have now implemented more advanced algorithms. In addition to VMC, Monaco, and AXB discussed above, GammaPlan also implemented a CT‐based, convolution algorithm to replace the traditional MR‐ and water‐based dose calculation. As such, most clinics have started adopting Type‐B and Type‐C dose algorithms for SRS. A few studies have been conducted to compare the calculated dose differences between different types of algorithms for cranial SRS^14^ on patients and phantoms.^34^ However, in these studies, despite confirming that Type‐C algorithms offer the most accurate dosimetry, target dose differences were usually relatively small for brain SRS cases, and larger magnitudes of dose discrepancies (3%–17%) were only seen for highly heterogeneous interfaces such as the bone‐tissue‐air interface of the sinus and the interface of the high‐density embolization material in the arteriovenous malformation (AVM) cases.^20^

In this context, our study reported provocative new results that large target dose differences of up to 15% can exist between the most advanced Type‐C algorithms for MLC‐based brain SRS, especially for smaller targets. This is even more relevant with MLC‐based SRS that has become increasingly prevalent, particularly with smaller targets such as commonly treated with SRS for multiple brain metastases. Our study is the first to compare two different Type‐C algorithms, and is to our knowledge one of the most comprehensive among all dose algorithm studies, with a very large number of patient cases (>100 plans and >400 targets vs. 10−40 plans commonly seen in this line of studies), including both VMAT and DCA approaches, and with both simulation and phantom measurement results. This study is also limited as it does not extend to investigate the quantitative evaluation of the fundamental cause of the observed discrepancies between the algorithms, which is beyond the scope of the current work.

In our investigation, quite astonishing results were found that >5% target dose differences can exist fairly often between the two Type‐C algorithms (12% among 443 targets in our investigation) and the dose difference can be as large as over 15%. For these two major treatment planning systems for LINAC‐based SRS and their respective most advanced dose algorithms, this could mean that a practice using Eclipse could be prescribing up to 15% hotter than another practice using Elements for some targets. We attempted to trend the cases with large (>5%) dose differences with a few different factors, including target size, planning and delivery technique, off‐axis (target‐to‐isocenter) distance, and heterogeneity (target‐to‐skull distance). Among these factors, only target size was found to associate with the occurrence of large dose differences. The two algorithms tend to agree well for larger targets. But for very small targets (target volume < 0.3 cm^3^ or equivalent sphere diameter < 8.3 mm), larger target dose differences > 5% are sometimes calculated between the two Type‐C algorithms.

While the exact causes of these large dose differences are beyond the scope of the current investigation, some possible contributors could include the modeling of the multi‐leaf collimators (MLCs) in the two algorithms and the resampling differences between the two planning systems. One recent study found that refinement of MLC modeling helps improve the gamma passing rates between AAA SRS/SBRT plans and their log‐file‐based patient‐specific quality assurance (QA) recalculation using a collapsed cone convolution superposition algorithm (Mobius Medical System, Houston, TX, USA).^35^ In that investigation, the MLC modeling parameters of the QA software were refined, and good agreement between the two Type‐B algorithms (<3%) was attainable for MLC fields bigger than 2 cm × 2 cm.

In our study, the two Type‐C algorithms were both thoroughly commissioned and validated, and benchmarked to the vendor‐provided representative data. However, there are some beam modeling differences between the two vendors. BrainLab requires beam data from MLC‐defined fields including very small field sizes (0.5 × 0.5 cm^2^), while Varian recommends those from jaw‐defined larger (3 × 3 cm^2^ and up) field sizes.^36^ Varian explains that depth dose and profile measurements for fields smaller than 3 × 3 cm^2^ are not considered in beam modeling, and additional small‐field output factors only affect jaw‐defined but not MLC‐defined fields in AXB. Moreover, the two TPSs also use different source models and different model parameter optimization. For our system, AXB has a source size of 1.5 mm and 0.0 mm in X and Y directions, while VMC has a primary source size of 0.9 mm in both X and Y, and a first scatter source size of 8.8 and 14.6 mm in X and Y directions. Nevertheless, Table 3 in this study shows that the two algorithms agree well even for small square jaw‐defined and MLC‐defined fields. Tables 3 and 4 together also show that VMC starts trending higher calculated dose than AXB as the field size gets smaller for MLC‐defined field, similar to what's observed in our patient and phantom studies.

Another possible cause is the fact that the two TPSs utilize different MLC modeling in the calculation, such as physical MLC modeling with rounded tip and tongue‐and‐groove structures in VMC, and dosimetric leaf gap (DLG), MLC transmission, and tongue thickness used for fluence calculation in AXB. VMC utilizes a physical shape of MLC modeling with a rounded tip in the photon interaction calculation, whereas AXB models DLG and tongue thickness in a simple shape to block fluence, compensating for slight transmission at the edge of MLCs. However, as shown in Table 3, scattering at the edge of the rounded tip and tongue could potentially redirect the transmitted photons with a small angle, which may increase the dose to the target center. For a very small field, the contribution of dose from the MLC edge increases, thereby amplifying the effect of MLC modeling, as shown in both Tables 3 and 4. We also investigated the impact of the DLG factor variation in Eclipse AXB dose calculation. The result showed that changing the DLG value from 0.042 cm (our clinical value) to 0.072 cm yielded only 1% in the target dose difference in our phantom case, indicating the DLG factor is unlikely a major contributor to the large target dose difference between AXB and VMC we observed in this study. One of the possible reasons for this small impact could be the fact that Elements plans are usually not very modulated.

Another recent study also reported non‐negligible dose‐volume metric differences between planning systems due to structure and dose grid resampling, especially for very small SRS targets, and called for better standardization.^37^ These factors, among others, could have contributed to the surprisingly large target dose differences we observed in our patient and phantom cases between VMC and AXB. This was observed in our homogeneous phantom experiment. In this experiment, the calculated dose was obtained using the mean dose of a contoured “effective volume of the ion chamber” that was separately sampled in the two treatment planning systems. For the smallest PTV (0.6 cm), the AXB recalculated 3064 cGy mean dose in the sensitive IC volume, while the VMC calculated mean dose in Elements and that resampled in Eclipse was 3590 cGy (17.2% higher than AXB) and 3336 cGy (8.9% higher than AXB), respectively. The 8.3% difference was primarily due to volume resampling. For larger PTVs, the changes were much smaller and less than 0.3%. This can be explained by the fact that the size of PTV (0.6 cm) was similar to the sensitive volume of the IC, the large dose change of the volume mean dose when simply read out in the two treatment planning systems was due to the structure sampling differences and the steep dose gradients around the small volume. However, the results from larger PTVs in this phantom experiment were not noticeably affected by the sampling effect. Moreover, in the large‐cohort patient study, the VMC and AXB dose was compared using a common platform by importing the Elements dose file into Eclipse. Structure sampling uncertainty did not play a role in the observed dose differences. Along with the head phantom experiment with film measurements where the sampling uncertainty also played no major role, the large relative dose differences between the two advanced dose algorithms are likely attributable to other factors.

While the accuracy of either algorithm has been benchmarked in various studies before their wide clinical adoption, the new findings from our study and the popular use of single‐isocenter‐multiple‐target SRS to treat increasingly smaller metastatic lesions warrant further investigation on and re‐examination of these commercial dose algorithms including the most advanced ones.

## 5 CONCLUSION

Utilizing a large patient cohort, clinical brain SRS dosimetry was compared between two advanced algorithms each used by a mainstream treatment planning system for LINAC‐based SRS. Ion chamber and film measurements were performed on a homogeneous phantom and an anthropomorphic phantom to benchmark calculated doses. Our findings indicate that large target dose differences of up to 15% can exist between the two algorithms for small targets and that VMC tends to have a slightly closer agreement with measurements than AXB. Among the investigated factors, small target sizes (<0.3 cm^3^) were found to associate with a higher chance of a >5% target dose difference between the two algorithms. Further investigation is warranted to better understand the discrepancy and improve dose calculation accuracy for modern SRS treatments.

## AUTHOR CONTRIBUTIONS

Hyunuk Jung and Dandan Zheng helped plan generation and data analysis. Sean Tanny, Olga Dona Lemus, and Dandan Zheng helped study design. Michael Milano, Sara Hardy and Kenneth Usuki reviewed the plan. All coauthors reviewed and edited the manuscript.

## CONFLICT OF INTEREST STATEMENT

The authors declare no conflicts of interest.
